# Hydrophobic Drug-Loaded PEGylated Magnetic Liposomes for Drug-Controlled Release

**DOI:** 10.1186/s11671-017-2119-4

**Published:** 2017-05-18

**Authors:** Andri Hardiansyah, Ming-Chien Yang, Ting-Yu Liu, Chih-Yu Kuo, Li-Ying Huang, Tzu-Yi Chan

**Affiliations:** 1 Department of Metallurgy and Materials Engineering, Bandung Institute of Technology and Science, Bekasi, 17530 Indonesia; 2 Department of Materials Science and Engineering, National Taiwan University of Science and Technology, Taipei, 10607 Taiwan; 3 Department of Materials Engineering, Ming Chi University of Technology, New Taipei City, 24301 Taiwan; 4 Institute of Polymer Science and Engineering, National Taiwan University, Taipei, 10617 Taiwan

**Keywords:** PEGylated liposomes, Curcumin, Magnetic nanoparticles, High-frequency magnetic field, Drug controlled release

## Abstract

Less targeted and limited solubility of hydrophobic-based drug are one of the serious obstacles in drug delivery system. Thus, new strategies to enhance the solubility of hydrophobic drug and controlled release behaviors would be developed. Herein, curcumin, a model of hydrophobic drug, has been loaded into PEGylated magnetic liposomes as a drug carrier platform for drug controlled release system. Inductive magnetic heating (hyperthermia)-stimulated drug release, in vitro cellular cytotoxicity assay of curcumin-loaded PEGylated magnetic liposomes and cellular internalization-induced by magnetic guidance would be investigated. The resultant of drug carriers could disperse homogeneously in aqueous solution, showing a superparamagnetic characteristic and could inductive magnetic heating with external high-frequency magnetic field (HFMF). In vitro curcumin release studies confirmed that the drug carriers exhibited no significant release at 37 °C, whereas exhibited rapid releasing at 45 °C. However, it would display enormous (three times higher) curcumin releasing under the HFMF exposure, compared with that without HFMF exposure at 45 °C. In vitro cytotoxicity test shows that curcumin-loaded PEGylated magnetic liposomes could efficiently kill MCF-7 cells in parallel with increasing curcumin concentration. Fluorescence microscopy observed that these drug carriers could internalize efficiently into the cellular compartment of MCF-7 cells. Thus, it would be anticipated that the novel hydrophobic drug-loaded PEGylated magnetic liposomes in combination with inductive magnetic heating are promising to apply in the combination of chemotherapy and thermotherapy for cancer therapy.

## Background

Science and technology in medicine still dealing and continue to develop the optimum strategies to inhibit and kill the cancerous cells. Common cancer therapy, including surgery, chemotherapy, and radiotherapy, still remain challenges due to the presence of various side effects related to the ineffectiveness treatment of those therapy. Thus, new strategy is in needed to overcome the serious obstacles in cancer treatment. Nanotechnology and nanomedicine offer new opportunity for cancer treatment. In this respect development of nanoparticles with various feature and functions along with the innovation of the cancer treatment methodology has been conducted experimentally in in vitro and in vivo [1, 2].

Liposome is one of the nanoparticles that have been widely used as a drug carrier for encapsulation of numerous drug and agents both for cancer or non-cancer treatment, which is a spherical bilayer membrane exhibited a well-developed of unique and important properties that needed for cancer therapy including a good biocompatibility, appropriate size, drug loading ability, and versatile surface functionalization [3, 4]. For instance, liposomes surfaces can be readily modified by tethering various substances with specific functions. Polyethylene glycol (PEG) could be attached into the liposomes surface in order to enhance the circulation time of liposomes in the bloodstream [5, 6]. Furthermore, liposomes vesicles with size approximately a hundred nanometers or less exhibited enhanced permeability and retention (EPR) effects which further develop liposomes as passively-targeted nanomaterial [7, 8]. The nanomaterial localization phenomena presented mainly in inflammation and cancer regions. However, a passive targeting of drug carriers at the cancerous site is not sufficient to obtain optimum therapeutic efficacy of the drug. Thus, the development of externally or internally active stimuli would gain an interesting role for promoting localization and action-in-pathological site [9].

According to the structure and morphology, liposomes were fabricated by the hydrophilic and hydrophobic region. Various drugs and agents have been encapsulated inside the region to develop some specific functions. In this respect, magnetic nanoparticles have been embedded into the liposomes, namely magnetic liposomes [3, 10–12] or magnetoliposomes [11, 13–16], to achieve specific functions in magnetic-related characteristic such as contrast agent [17], magnetic-targeted ability[18], and heating generation [3, 19]. Specifically, through the guidance of external magnetic field, magnetic liposomes could be directed into the specific area of tumor cells, then promote another specific function, including drug release [3, 16, 20, 21] and killing the cancerous cells [3, 13, 16, 22, 23]. High-frequency magnetic field (HFMF) has been developed as a system to assist the magnetic-based nanoparticles developed the specific function based on the interaction between the magnetic-based nanoparticles and HFMF exposure [1–3, 24, 25].

Chemotherapeutic drug has an important function in the diseases treatment, such as cancer therapy. However, the common chemotherapeutic cancer drugs, such as doxorubicin, exhibited toxicity and serious adverse effects [26]. Thus, the development of therapeutic agents or drugs with no side effects to the normal cells is in needed as an important strategy in the treatment of cancer or tumor cells [27]. Recently, a number of natural-based compounds have been investigated. Curcumin, a natural phenolic compound have attracted a numerous multidisciplinary researchers in natural medicine, food technology, and biomaterials science [1], and has been commonly used as a traditional medicine and additive ingredients for foods. For the chemotherapeutic properties, curcumin exhibited beneficial properties, including antioxidant, anti-inflammatory, antimicrobial, anticancer, and wound healing characteristics [27]. Curcumin has been demonstrated to inhibit proliferation of cancer cell and to induce apoptosis without promoting adverse effects [28]. The characteristic of curcumin reported against various cancer cells indicate its ability to affect different targets through their interference in various cellular mechanisms [29]. However, the utilization of curcumin for further applications has been limited due to its low aqueous solubility properties and low systemic bioavailability. Previous studies revealed that the detection of curcumin concentration in serum was extremely low although a high concentration of curcumin has been orally-administered [30]. Recently, researchers have also been combined curcumin into the various features of nanomaterial to enhance the water solubility, thereby increasing its circulation time and bioavailability thus enhance its ability to target the cancerous cells [1, 28, 30–34].

In the present study, liposome-based drug carrier would be developed by encapsulation of oil-phase magnetic nanoparticles and curcumin in the polyethylene glycol-modified liposomes (PEGylated liposomes). The structural and morphology characterizations, high-frequency magnetic field (HFMF)-induced drug release, in vitro cellular cytotoxicity and cellular internalization-induced by magnetic guidance would be investigated.

## Methods

### Materials

Synthetic lipid 1,2-dipalmitoyl-*sn*-*glycero*-3-phosphocholine (DPPC) (purity > 99%) and 1,2-distearoyl-*sn*-*glycero*-3-phosphoethanolamine-*N*-[carbonyl methoxy(polyethylene glycol)-2000 were purchased from Avanti polar lipid, AL, USA. Cholesterol, curcumin, 1,10-dioctadecy-3,3,30,30-tetramethylindocarbocyanine perchlorate (Dil), ferric chloride tetrahydrate (FeCl_2_.4H_2_O), 4′,6-diamidino-2-phenylindole (DAPI), oleic acid and chloroform were purchased from Sigma-Aldrich, St. Louis, MO, USA. Ferric chloride hexahydrate (FeCl_3_.6H_2_O) was purchased from Shimakyu’s Pure Chemical, Osaka, Japan. Ethanol (95%) was purchased from Acros, USA. For the cell culture experiments, fibroblasts (L-929) cells were obtained from ATCC CRL-1503TM and human breast cancer (MCF-7) cells were obtained from Food Industry Research and Development Institute (Taiwan). Dulbecco’s modified Eagle’s medium-high glucose (DMEM), trypsin, dimethylsulfoxide (DMSO), trypan blue, and 3-(4,5-dimethylthiazo-2-yl)-2,5-diphenyl tetrazolium bromide (MTT) powder were purchased from Sigma Aldrich, St. Louis, MO, USA. Fetal bovine serum (FBS) was purchased from BD Biosciences, San Jose, CA, USA. High-purity water purified by a Milli Q Plus water purifier system (Milipore, USA), with a resistivity of 18.3 MΩcm was used in all experiments. All the chemicals were used without further purification.

### Preparation of Curcumin-Loaded PEGylated Magnetic Liposomes

Liposomes-based drug carrier were prepared through the well-established thin-film hydration method followed by extrusion techniques as the method described previously with minor modification [3, 4]. Briefly, 1,2-dipalmitoyl-*sn*-*glycero*-3-phosphocholine (DPPC): cholesterol: 1,2-distearoyl-*sn*-glycero-3-phosphoethanolamine *N* [carbonyl-methoxy (polyethylene glycol)-2000 were mixed at a composition of 80:20:5 mol%. Oleic acid coated magnetic nanoparticles (OAMNP) have been prepared via co-precipitation method (Supplementary Information). Lipid mixtures, curcumin, and OAMNP were dissolved homogeneously in chloroform: methanol mixture (3:1 *v/v*) then subjected into rotary evaporation system (N-1200 series, Eyela®, Tokyo Rikakikai Co., Ltd., Tokyo, Japan), thus resulting a thin dry lipid film. Hydration process of thin dry lipid film was accomplished by adding PBS pH 7.4 at 60 °C for 1 h then subsequently placed to a bath-type sonicator for harvesting the resulting liposomes. Un-encapsulated magnetic nanoparticles were separated through 1000*g* centrifugation for 15 min and magnetic separation [23]. Afterward, the resultant liposomes were homogenized using ultrasonicator (Probe-type sonicator, VCX 750, Vibra-Cell ^TM^, SONICS®, Sonics and Materials, Inc., Newton, CT, USA). Eventually, the suspension was extruded several times through a 0.22-μm filter to reduce the size and for sterilization. The resulting product was termed as curcumin-loaded PEGylated magnetic liposomes and then stored at 4 °C prior to characterizations. Liposomes uptake was visualized by using fluorescent Dil marker [31]. Empty liposomes without curcumin and oleic acid-coated magnetic nanoparticles were used as a control.

### Characterizations

Structure and morphology of curcumin-loaded PEGylated magnetic liposomes were characterized by transmission electron microscopy, TEM-7650, Hitachi, Chiyoda-ku, Japan. Prior to the TEM observation, an aliquot of suspension of samples was diluted with water until optically clear. Phosphotungstic acid (PTA) was used as the staining agent for PEGylated liposomes. For OAMNP and curcumin-loaded PEGylated magnetic liposomes, TEM imaging was conducted without using PTA. The samples were not stained as the magnetic nanoparticles can be visualized directly due to their high electron density [35]. Furthermore, the average particle size and zeta (ζ) potential of the sample were determined at 25 °C and pH 7.4 by using dynamic light scattering (DLS) spectrophotometer, Horiba Instrument, Horiba, Kyoto, Japan with helium-neon laser with wavelength of 633 nm, scattering angle of 90°, and refractive index of 1.33 at 25 °C. Zeta (ζ) potential was determined with the same apparatus with DLS through electrophoretic mobility measurement and calculated using Helmholtz-Smoluchowski’s equation.

### Inductive Magnetic Heating by HFMF

Inductive magnetic heating (hyperthermia) experiment was conducted by using high-frequency magnetic field (HFMF) system as the method reported previously with minor modification [1, 3, 36]. Briefly, the samples were positioned to the center of copper coil in the HFMF generator for 30 min. The change of the temperature was recorded by an alcohol thermometer. PBS was used as a control. Each experiment was performed triplicate.

### Magnetic Characterizations

The magnetization study was conducted to evaluate the magnetic characteristics of the synthesized PEGylated magnetic liposomes in response to an externally applied magnetic field stimuli based on the method described with minor modification [10]. 1,10-dioctadecy-3,3,30,30-tetramethylindocarbocyanine perchlorate (Dil) was used as a fluorescent marker. Briefly, an aliquot of Dil-loaded PEGylated magnetic liposomes was diluted with PBS. An aliquot of the diluted sample was placed on a glass slide and positioned at a certain distance among permanent magnetic field. Fluorescent images (fluorescence microscope, Olympus, Japan) of the samples at certain interval times was taken to define the movement of the formulation along the direction of applied magnetic field. Magnetization as a function of the field were also evaluated using a vibrating sample magnetometer (VSM) Lakeshore model 7400 at room temperature.

### Encapsulation Efficiency and In Vitro Drug Release Studies

Encapsulation efficiency defined as the ratio of encapsulated drug to the total drug in the system. Briefly, samples of complete liposomes preparation are centrifuged at 10,000*g* for 15 min and the absorbance of the clear supernatant was measured by UV-Vis spectroscopy at 425 nm. The encapsulation efficiency was calculated as [37, 38]:

Encapsulation efficiency (%) = total drug-total free drug/total drug × 100%

Calibration curve was obtained by plotting absorbance of a serial dilution of curcumin from 2 to 20 μg mL^−1^ at 425 nm using UV spectroscopy. A linear equation was fitted as *A* = 0.1534C + 0.0447, *R* ^2^ = 0.991, where A is absorbance and C is the drug concentration.

The curcumin release profile from curcumin-loaded PEGylated magnetic liposomes was determined by dialysis method. The curcumin release study was carried out at temperatures of 37 °C and 45 °C. 0.5% tween-80 with 20% ethanol (*v*/*v*) in PBS was used as receptor medium [39]. Briefly, 1 mL of the suspensions were dialyzed against 20 mL receptor medium. At certain time intervals, 1 mL of receptor medium was taken out for analysis and fresh receptor medium solution was replenished and its concentration of the released drugs was measured by UV spectroscopy at 425 nm. The cumulative release was calculated as follows [33]:

$$ \mathrm{Cumulative}\ \mathrm{release}\ \left(\%\right) = {R}_t/ D \times 100\% $$

where *D* and *R*_*t*_ represent the initial amount of curcumin loaded and the cumulative amount of curcumin released at time *t*, respectively. Each experiment was performed three times. Furthermore, Curcumin release behavior from the curcumin-loaded PEGylated magnetic liposomes was also conducted by applying externally HFMF exposure to elaborate the mechanism of inductive magnetic heating (hyperthermia)-triggering. Briefly, the samples were placed under HFMF for 30 min. An aliquot of curcumin was taken from the test tube during the test for 10 min first and continued 10 min until 30 min during HFMF exposure. The quantity of curcumin was quantified as described by the aforementioned method.

### Cell Culture

The cells cultures of fibroblast (L-929) and MCF-7 cells were conducted through the incubation of the cells under saturated humid conditions at 37 °C with 5% CO_2_. The cells were cultured with DMEM containing 10 vol.% fetal bovine serum (FBS) and 1 vol.% antibiotic antimycotic solution. Medium was changed every day until reaching approximately 70 to 80% confluency.

### 3-(4,5-dimethylthiazo-2-yl)-2,5-Diphenyl Tetrazolium Bromide (MTT) Assay

Cell growth and cytotoxicity were determined using MTT assay. In 96 well plates, L-929 and MCF-7 cells (10,000 cells per well) were cultured in each well. These plates were divided into several groups and incubated under saturated humid conditions at 37 °C and 5% CO_2_. After 24 h of incubation, the medium was replenished. Among these plates, some plates were added with 200 μL of PEGylated liposomes or PEGylated magnetic liposomes. After culturing for 48 h, MTT solution were added to each well. After incubating for another 3 h at 37 °C, the medium was withdrawn and replaced with 200 μL DMSO and allowed to stand about 15 min for complete reaction. Furthermore, the plates were shaken, and the readings were taken at 570 nm using an ELISA reader (Sunrise, Tecan, Männedorf, Switzerland). Proliferation or viability of the cells was calculated as follows: Proliferation (%) = Ac/A_0_ × 100%. Cytotoxicity of curcumin-loaded PEGylated magnetic liposomes was conducted through aforementioned procedure with the variation of curcumin concentration against MCF-7 cells.

### Intracellular Uptake and Fusion

The intracellular uptake and fusion behavior of PEGylated magnetic liposomes formulations toward MCF-7 cell was performed using 1,10-dioctadecy-3,3,30,30-tetramethylindocarbocyanine perchlorate (Dil) as a fluorescent marker. Dil is a lipophilic dye with an orange-red-fluorescence and its liposomes encapsulation characteristic similar to curcumin [31]. Magnetic targeting experiments were conducted as method described previously with minor modification [40]. Briefly, MCF-7 cells (20,000 cells/well) were cultured in each well of 4-well Nunc Lab-Tek chamber slides (Thermo Scientific, Rochester, New York, USA) and incubated at 37 °C in a 5% CO_2_ incubator for 24 h. Further, cells were incubated with Dil-loaded PEGylated magnetic liposomes and then exposed in the presence of external magnets (neodymium-based magnets with the magnetic strength: 26-50 MGOe, Taiwan) for 3 h. As control, cells were also incubated with PEGylated liposomes, PEGylated magnetic liposomes, Dil-loaded PEGylated liposomes and Dil-loaded PEGylated magnetic liposomes, but exposed in the absence of external magnets. After incubation, cells were washed several times with PBS and fixed with 4 wt% paraformaldehyde for 10 min. Further, the fixing solution was aspirated and traces of fixing agents were removed by rinsing several times with PBS. Cells were then stained using 4′,6-diamidino-2-phenylindole (DAPI) for 10 min. Eventually, the staining solution was aspirated and traces of staining agent were removed by rinsing several times with PBS. Slides were mounted using a Vectashield mounting medium (H-1000), Vector Laboratories Inc., (Burlingame, California, USA). The cellular internalization and fusion behavior were observed under fluorescence microscope (Olympus, Japan).

## Results and Discussion

### Morphology and Characterization

**Fig. 1:**
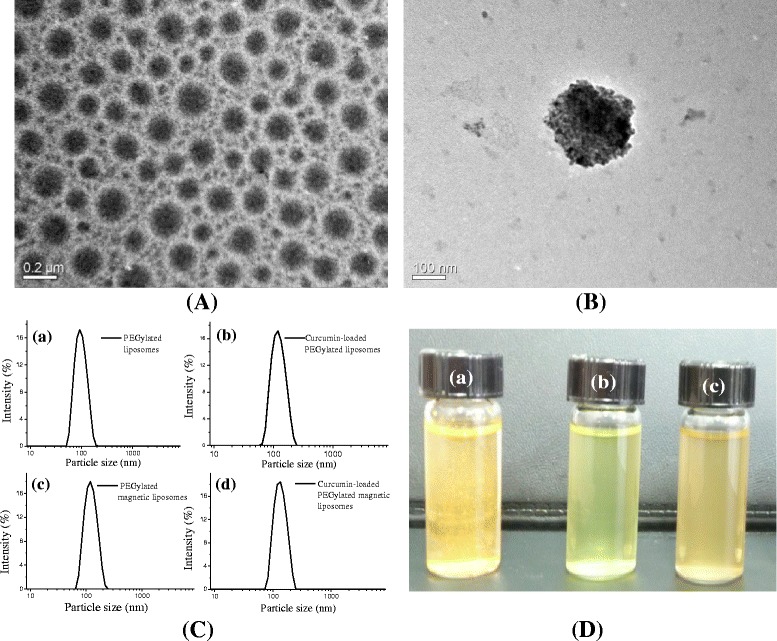
TEM images of (**a**) curcumin-loaded PEGylated liposomes and (**b**) curcumin-loaded PEGylated magnetic liposomes, (**c**) particle size distribution of various formulations of PEGylated liposomes formulations; (**d**) images of curcumin solution (**a**), curcumin-loaded PEGylated liposomes (**b**) and curcumin-loaded PEGylated magnetic liposomes (**c**)

TEM observation confirmed that the oleic acid-coated magnetic nanoparticles (OAMNP) exhibited the spherical morphology of nanoparticles with the average particle size was around 10 nm (Supplementary information S1a). X-ray diffraction (XRD) analysis of OAMNP revealed that the crystalline phases of iron oxide nanoparticles are similar with the magnetite (JCPDS 19–0629) (Supplementary information S1b). TEM observation confirmed that the curcumin-loaded PEGylated liposomes and curcumin-loaded PEGylated magnetic liposomes were developed in the spherical structure (Fig. 1a, b) with the average particle size was around 100 nm, which is appropriate for EPR effects for targeting tumor cells. According to the DLS measurement, the particle size of curcumin-loaded PEGylated liposomes and curcumin-loaded PEGylated magnetic liposomes are about 120–140 nm (Fig. 1c). The solubility of curcumin in water is limited because the hydrophobic characteristic of curcumin (Fig. 1d-a). On the other hand, the curcumin-loaded PEGylated liposomes (Fig. 1d, b) and curcumin-loaded PEGylated magnetic liposomes (Fig. 1d, c) shows a good dispersion in water system. This proved the successful encapsulation of curcumin and oleic acid magnetic nanoparticles in the liposomes compartment. Encapsulation efficiency of curcumin in PEGylated liposomes and PEGylated magnetic liposomes was about 78.06 ± 0.57% and 76.15 ± 1.6%, respectively. In this respect, the hydrophobic lipid bilayer compartment of liposomes provide the region for the encapsulation of curcumin [28].

The colloidal stability, dispersion system, and the interaction of nanoparticles with cells are related with the electric charge of the particle surface which is represent by zeta (ζ) potential. For the reference, the zeta potential of non-PEGylated liposomes display the negative charge of −17 mV. On the other hand, the zeta potential of PEGylated liposomes and curcumin-loaded PEGylated magnetic liposomes increased to −2.86 and −3.17 mV, respectively. The increased zeta potential indicated the charge shielding effect of polyethylene glycol (PEG) and curcumin. These characteristics prevent liposomes to be fusion and aggregation to enhance the colloidal stability simultaneously [31, 41].

### Magnetic Properties

**Fig. 2:**
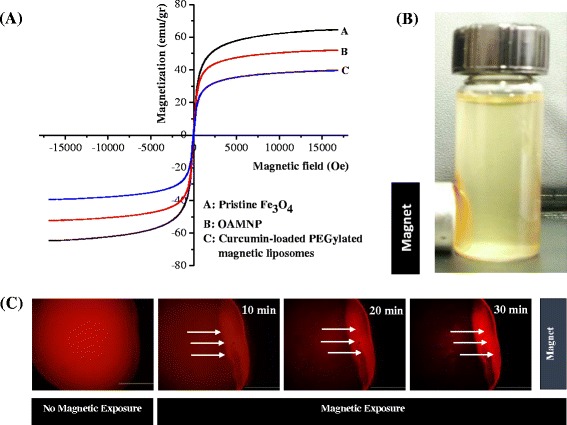
(**a**) Hysteresis curve of Fe_3_O_4_ (MNP), oleic acid-coated Fe_3_O_4_ (OAMNP) and curcumin-loaded PEGylated magnetic liposomes, (**b**) the interaction of curcumin-loaded PEGylated magnetic liposomes with external magnetic field and (**c**) magnetic attraction behavior of Dil-loaded PEGylated magnetic liposomes towards external magnetic field exposure by florescence microscopy (scale bar: 100 μm)

The magnetic properties of the oleic acid-coated magnetic nanoparticles (OAMNP) and curcumin-loaded PEGylated magnetic liposomes magnetic were evaluated through vibrating sample magnetometer (VSM) at room temperature. Figure 2a shows the hysteresis curve of OAMNP and curcumin-loaded PEGylated magnetic liposomes, indicated the superparamagnetic properties of nanoparticles. The saturation magnetization values of pristine Fe_3_O_4_, OAMNP and curcumin-loaded PEGylated magnetic liposomes were 64.66, 54.00, and 39.72 emu/g, respectively. Superparamagnetic properties of curcumin-loaded PEGylated magnetic liposomes are important for biomedical applications to prevent aggregation and enable rapid dispersal in the absence of magnetic field. The decreasing of magnetization values of curcumin-loaded magnetic liposomes might be due to the modification of non-magnetic phospholipids bilayers. The lipid bilayer interferes the domain alignment and inhibited the interaction of the OAMNP encapsulated within the lipid bilayers to the external magnets (neodymium-based magnets) exposure [28, 42]. Figure 2b shows that the curcumin-loaded PEGylated magnetic liposomes could interact easily with the external magnets exposure.

Figure 2c shows the magnetic movement of Dil-loaded PEGylated magnetic liposomes triggered in the external magnets exposure by florescence microscopy. A homogeneous distribution of Dil-loaded PEGylated magnetic liposomes were monitored in the absence of external magnets. Upon application of external magnets exposure, the Dil-loaded PEGylated magnetic liposomes rapidly moved towards the magnet as a function of times. These characteristics clearly demonstrate that the formulations could attracted to the external magnets exposure.

### Inductive Magnetic Heating Ability

**Fig. 3:**
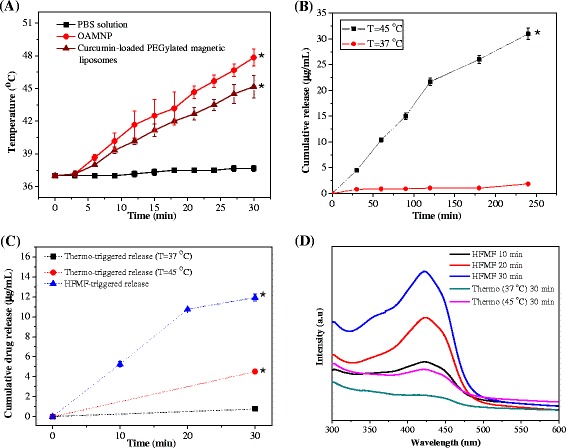
(**a**) Inductive heating ability of the oleic acid-coated magnetic nanoparticles (OAMNP) and the curcumin-loaded PEGylated magnetic liposomes under HFMF exposure. PBS solution was used as a control sample (*p < 0.05), (**b**) cumulative drug release profile of curcumin-loaded PEGylated magnetic liposomes at various temperature (* *p* < 0.05 against the sample incubated at 37 °C), (**c**) cumulative drug release of curcumin-loaded PEGylated magnetic liposomes under HFMF exposure (*p < 0.05) and (**d**) curcumin release profile monitored by UV-Visible spectroscopy

Figure 3a shows the results of inductive heating of curcumin-loaded PEGylated magnetic liposomes under high-frequency magnetic field (HFMF) exposure. The results showed that, the temperature increased to 45 °C and 48 °C for the curcumin-loaded PEGylated magnetic liposomes and OAMNP, meanwhile the control sample (PBS solution) exhibited no significant inductive magnetic heating. This temperature difference was related to the inductive magnetic heating effect generated from the magnetic nanoparticles in the presence of HFMF.

### In Vitro Drug Release Studies

Dialysis method was conducted to investigate the drug release profile of curcumin from PEGylated magnetic liposomes at various temperature. As shown in Fig. 3b, curcumin release from curcumin-loaded PEGylated magnetic liposomes was only ~ 2 μg mL^−1^ after 4 h incubation at 37 °C. Curcumin release from curcumin-loaded PEGylated magnetic liposomes was increased significantly to ~30 μg mL^−1^ after 4 h incubation at 45 °C, which arrived 15 times difference. This result indicated that curcumin-loaded PEGylated magnetic liposomes have desirable thermo-sensitivity ability. The release of the curcumin molecules could be attributed to the disruption of the membrane lipid bilayer at elevated temperatures, thereby releasing curcumin to the surrounding environment simultaneously. In these respect, the structure and fluidity of the lipid bilayers are greatly influenced by the phase transition temperature which further affects the release of curcumin from the curcumin-loaded PEGylated magnetic liposomes. In this respect, physiological temperature (37 °C) is below the transition temperature of phosphatidylcholine as the main structural component of these liposomes, thus the release of curcumin in physiological environments is inhibited. However, a hyperthermia temperature of 45 °C is above the transition temperature, thus increasing the release of curcumin through the structural disruption of lipid bilayer [28].

Figure 3c shows the cumulative drug release of curcumin-loaded PEGylated magnetic liposomes under HFMF exposure and curcumin release profile monitored by UV-visible spectroscopy (Fig. 3d). The cumulative curcumin release from curcumin-loaded PEGylated magnetic liposomes with HFMF treatment arrived to ~12 μg mL^−1^ after 30 min of HFMF exposure. Meanwhile, cumulative release of curcumin from curcumin-loaded PEGylated magnetic liposomes after incubated at 37 and 45 °C for 30 min were only 0.8 and 4.5 μg mL^−1^, respectively. These phenomena might be related to the incorporation of oleic acid-coated magnetic nanoparticles in the curcumin-loaded PEGylated magnetic liposomes which generate localized heating under HFMF stimulus, followed by increasing the permeability of lipid bilayer, thus enhanced the curcumin release from liposomes compartment [25, 43].

### Cytotoxicity Studies

**Fig. 4:**
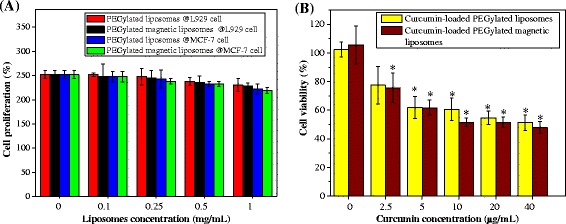
(**a**) Cell proliferation (%) of fibroblast (L-929) and human breast cancer (MCF-7) cell after incubation with various concentration of PEGylated liposomes and PEGylated magnetic liposomes for 48 h and (**b**) Cell viability (%) of MCF-7 cells treated with curcumin-loaded PEGylated liposomes and curcumin-loaded PEGylated magnetic liposomes with various concentrations for 48 h

MTT assay was conducted to evaluate the cellular cytotoxicity of PEGylated liposomes and PEGylated magnetic liposomes toward fibroblast (L-929) and human breast cancer (MCF-7) cells, thereby indicating the effect of those drug carriers on the growth and proliferation of cells. Based on the cell proliferation of L-929 and MCF-7 cells (Fig. 4a), the cells proliferate well in the incubation with PEGylated liposomes and PEGylated magnetic liposomes. This might be due to the better biocompatibility of the liposome-based system with the cell compartment [28, 33, 42, 44, 45]. These results confirmed that the drug carriers exhibited no cytotoxicity against L-929 cell and MCF-7 cells, suggesting good biocompatibility of the drug carriers. It is a potential to use the novel magnetic carriers to deliver the chemotherapeutic drugs in aqueous environment without harming the healthy cells.

### In Vitro Chemotherapy

We also evaluated the in vitro anticancer effects of these curcumin-loaded PEGylated magnetic liposomes using MTT assay. In this respect, curcumin-loaded PEGylated magnetic liposomes with various concentrations of curcumin have been prepared. As shown in Fig. 4b, the cytotoxicity of the curcumin-loaded PEGylated magnetic liposomes exhibited curcumin concentration-dependent manners. With the increase of curcumin concentration, the cytotoxicity of the curcumin-loaded magnetic liposomes was increased. These results showed the chemotherapeutic effects of curcumin to the cancer cells. Our results are in accordance with previous investigation that curcumin could induce apoptosis in cancerous cells [28, 33, 46]. Previous investigators also revealed that apoptosis could be generated through the generation of reactive oxygen species which sensitizing the cells into curcumin [46]. Eventually, this result offers the opportunity and advantages of the development of natural hydrophobic drug as a modality to treat cancerous cells without harming into the normal cells.

### Cellular Internalization and Magnetic Targeting

**Fig. 5:**
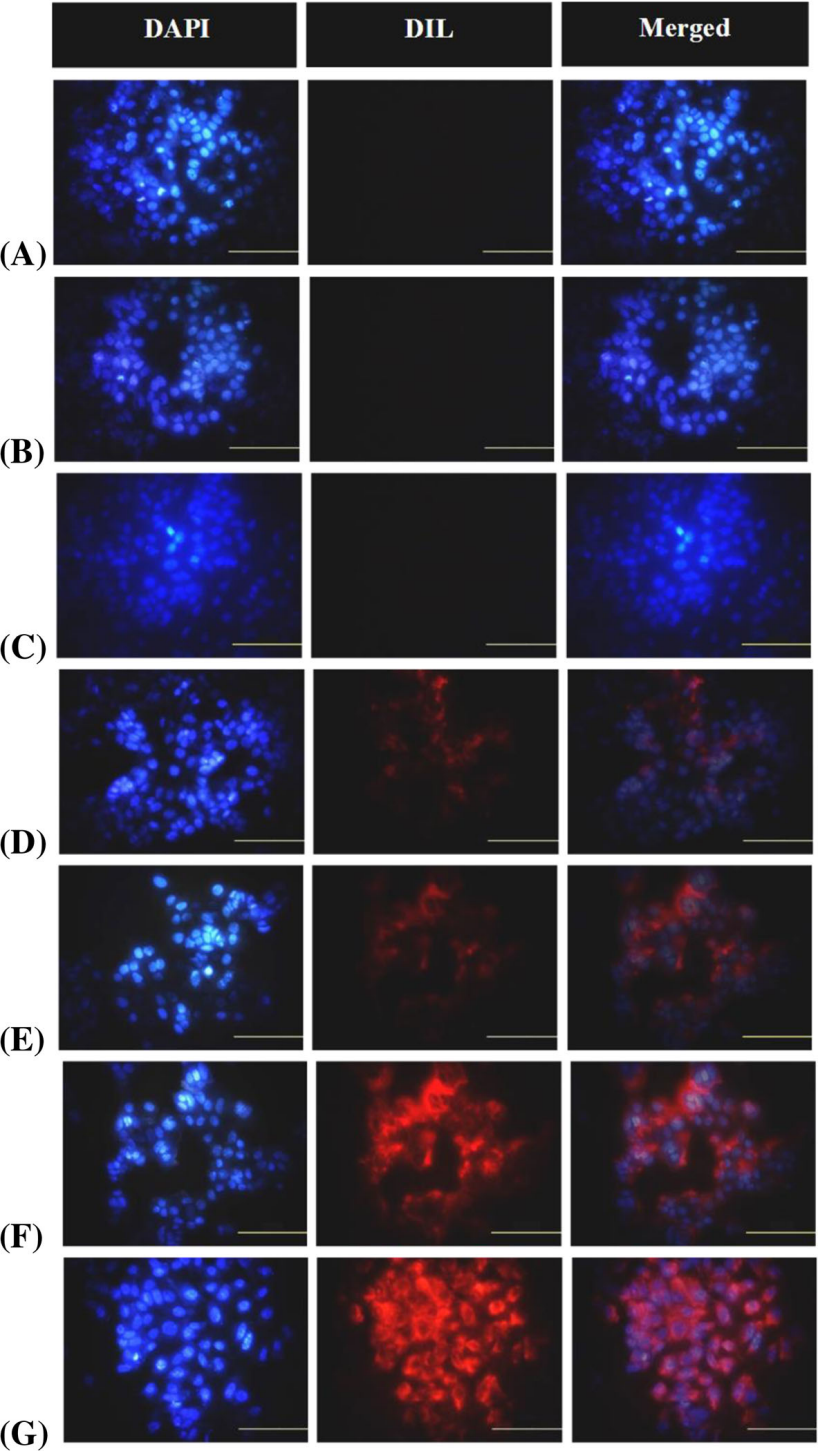
Fluorescence microscopy images of (**a**) human breast cancer (MCF-7) cells incubated in medium as control, (**b**) PEGylated liposomes, (**c**) PEGylated magnetic liposomes, and (**d**) Dil-loaded PEGylated liposomes without external magnets exposure; (**e**) Dil-loaded PEGylated liposomes, (**f**) 0.01 M of Dil-loaded PEGylated magnetic liposomes, and (**g**) 0.1 M of Dil-loaded PEGylated magnetic liposomes with external magnets exposure

Figure 5 shows the fluorescence images of the resultant drug carriers toward human breast cancer (MCF-7) cells compartment. Based on the DAPI staining, there is no significant different in the nucleus of cells treated with medium (Fig. 5a), PEGylated liposomes (Fig. 5b) and PEGylated magnetic liposomes (Fig. 5c), which further confirmed the excellent biocompatibility of the liposomes-based system. Dil-loaded PEGylated liposomes without (Fig. 5d) and with (Fig. 5e) magnetic field exposure also exhibited cellular internalization, based on the presence of Dil signal in the cellular compartment. Those phenomena might be due to the passive targeting mechanism of liposomes to the cellular compartment then followed by the diffusion or endocytosis mechanism into the cellular compartment. Furthermore, 0.01 M Dil-loaded PEGylated magnetic liposomes-treated cells (Fig. 5f) and 0.1 M Dil-loaded PEGylated magnetic liposomes-treated cells (Fig. 5g) with external magnets exposure exhibited cellular targeting. It was more pronounced in the influence of external magnets exposure, as confirmed by the highly Dil fluorescence signal around the cytoplasm and nucleus of the cells, exhibited concentration dependent. Eventually, these results confirmed the targeting activity of PEGylated magnetic liposomes could more pronounce and guided effectively by external magnets exposure. Magnetically-drug targeting effects might also promote the drug accumulation in targeted tumor site under magnetic field guidance which further increase the therapy effects of the drug for inhibiting cancer proliferation.

## Conclusions

In this study, we reported the development of PEGylated magnetic liposomes as drug vesicles for controlled releasing of curcumin by inductive magnetic heating. The magnetic drug carrier encapsulated natural hydrophobic anti-cancer drug, curcumin, exhibited an excellent stability and dispersed homogeneously in aqueous system. The releasing rate of curcumin from the drug carriers were manipulated through high-frequency magnetic field (HFMF) exposure, which might be applied for the cancerous cells (MCF-7 cells) therapy due to the enormous curcumin releasing. Fluorescence microscope observation revealed that the magnetic drug carriers could target effectively into the cellular compartment. Eventually, the magnetic drug carriers offer the potential application for cancer therapy through combination between natural hydrophobic drug, inductive magnetic heating (hyperthermia)-triggering releasing system, and magnetic-based targeting system.

## References

[1] Kuo C-Y, Liu T-Y, Chan T-Y, Tsai S-C, Hardiansyah A, Huang L-Y, Yang M-C, Lu R-H, Jiang J-K, Yang C-Y (2016). Magnetically triggered nanovehicles for controlled drug release as a colorectal cancer therapy. Colloids Surf B: Biointerfaces.

[2] Kuo C-Y, Liu T-Y, Hardiansyah A, Lee C-F, Wang M-S, Chiu W-Y (2014). Self-assembly behaviors of thermal- and pH- sensitive magnetic nanocarriers for stimuli-triggered release. Nanoscale Res Lett.

[3] Hardiansyah A, Huang LY, Yang MC, Liu TY, Tsai SC, Yang CY, Kuo CY, Chan TY, Zou HM, Lian WN, Lin CH (2014). Magnetic liposomes for colorectal cancer cells therapy by high-frequency magnetic field treatment. Nanoscale Res Lett.

[4] Hardiansyah A, Huang L-Y, Yang M-C, Purwasasmita BS, Liu T-Y, Kuo C-Y, Liao H-L, Chan T-Y, Tzou H-M, Chiu W-Y (2015). Novel pH-sensitive drug carriers of carboxymethyl-hexanoyl chitosan (chitosonic[registered sign] acid) modified liposomes. RSC Adv.

[5] Klibanov AL, Maruyama K, Beckerleg AM, Torchilin VP, Huang L (1991). Activity of amphipathic poly(ethylene glycol) 5000 to prolong the circulation time of liposomes depends on the liposome size and is unfavorable for immunoliposome binding to target. Biochim Biophys Acta Biomembr.

[6] Allen C, Dos Santos N, Gallagher R, Chiu GN, Shu Y, Li WM, Johnstone SA, Janoff AS, Mayer LD, Webb MS, Bally MB (2002). Controlling the physical behavior and biological performance of liposome formulations through use of surface grafted poly(ethylene glycol). Biosci Rep.

[7] Abraham SA, Waterhouse DN, Mayer LD, Cullis PR, MaddenTD, Bally MB (2005) The liposomal formulation of doxorubicin. Methods in enzymology, edition. San Diego: Elsevier Academic Press 391:71–97 10.1016/S0076-6879(05)91004-5 15721375

[8] El Maghraby GM, Barry BW, Williams AC (2008). Liposomes and skin: from drug delivery to model membranes. Eur J Pharm Sci.

[9] Sawant RR, Torchilin VP (2010). Liposomes as ‘smart’ pharmaceutical nanocarriers. Soft Matter.

[10] Nahar K, Absar S, Patel B, Ahsan F (2014). Starch-coated magnetic liposomes as an inhalable carrier for accumulation of fasudil in the pulmonary vasculature. Int J Pharm.

[11] Marie H, Lemaire L, Franconi F, Lajnef S, Frapart Y-M, Nicolas V, Frébourg G, Trichet M, Ménager C, Lesieur S: Superparamagnetic Liposomes for MRI Monitoring and External Magnetic Field-Induced Selective Targeting of Malignant Brain Tumors. Adv Funct Mater.2015:n/a-n/a.

[12] Di Corato R, Béalle G, Kolosnjaj-Tabi J, Espinosa A, Clément O, Silva AKA, Ménager C, Wilhelm C (2015). Combining magnetic hyperthermia and photodynamic therapy for tumor ablation with photoresponsive magnetic liposomes. ACS Nano.

[13] Yoshida M, Sato M, Yamamoto Y, Maehara T, Naohara T, Aono H, Sugishita H, Sato K, Watanabe Y (2012). Tumor local chemohyperthermia using docetaxel-embedded magnetoliposomes: interaction of chemotherapy and hyperthermia. Eur J Gastroenterol Hepatol.

[14] Bolfarini GC, Siqueira-Moura MP, Demets GJ, Morais PC, Tedesco AC (2012). In vitro evaluation of combined hyperthermia and photodynamic effects using magnetoliposomes loaded with cucurbituril zinc phthalocyanine complex on melanoma. J Photochem Photobiol B Biol.

[15] Qiu D, An X (2013). Controllable release from magnetoliposomes by magnetic stimulation and thermal stimulation. Colloids Surf B: Biointerfaces.

[16] Guo H, Chen W, Sun X, Liu Y-N, Li J, Wang J (2015). Theranostic magnetoliposomes coated by carboxymethyl dextran with controlled release by low-frequency alternating magnetic field. Carbohydr Polym.

[17] Martina MS, Fortin JP, Menager C, Clement O, Barratt G, Grabielle-Madelmont C, Gazeau F, Cabuil V, Lesieur S (2005). Generation of superparamagnetic liposomes revealed as highly efficient MRI contrast agents for in vivo imaging. J Am Chem Soc.

[18] Kubo T, Sugita T, Shimose S, Nitta Y, Ikuta Y, Murakami T (2000). Targeted delivery of anticancer drugs with intravenously administered magnetic liposomes in osteosarcoma-bearing hamsters. Int J Oncol.

[19] Yanase M, Shinkai M, Honda H, Wakabayashi T, Yoshida J, Kobayashi T (1998). Intracellular hyperthermia for cancer using magnetite cationic liposomes: an in vivo study. Jpn J Cancer Res.

[20] Babincová M, Čičmanec P, Altanerová V, Altaner Č, Babinec P (2002). AC-magnetic field controlled drug release from magnetoliposomes: design of a method for site-specific chemotherapy. Bioelectrochemistry.

[21] Tai LA, Tsai PJ, Wang YC, Wang YJ, Lo LW, Yang CS (2009). Thermosensitive liposomes entrapping iron oxide nanoparticles for controllable drug release. Nanotechnology.

[22] Kulshrestha P, Gogoi M, Bahadur D, Banerjee R (2012). In vitro application of paclitaxel loaded magnetoliposomes for combined chemotherapy and hyperthermia. Colloids Surf B: Biointerfaces.

[23] Peng Z, Wang C, Fang E, Lu X, Wang G, Tong Q (2014). Co-delivery of doxorubicin and SATB1 shRNA by thermosensitive magnetic cationic liposomes for gastric cancer therapy. PLoS One.

[24] Hu S-H, Liu T-Y, Liu D-M, Chen S-Y (2007). Controlled pulsatile drug release from a ferrogel by a high-frequency magnetic field. Macromolecules.

[25] Liu TY, Hu SH, Liu KH, Shaiu RS, Liu DM, Chen SY (2008). Instantaneous drug delivery of magnetic/thermally sensitive nanospheres by a high-frequency magnetic field. Langmuir.

[26] Rahman A, More N, Schein PS (1982). Doxorubicin-induced chronic cardiotoxicity and its protection by liposomal administration. Cancer Res.

[27] Salem M, Rohani S, Gillies ER (2014). Curcumin, a promising anti-cancer therapeutic: a review of its chemical properties, bioactivity and approaches to cancer cell delivery. RSC Adv.

[28] Nigam S, Kumar A, Thouas GA, Bahadur D, Chen Q (2013). Curcumin delivery using magnetic liposomes. J Nanopharmaceutics Drug Delivery.

[29] Anand P, Sundaram C, Jhurani S, Kunnumakkara AB, Aggarwal BB (2008). Curcumin and cancer: an “old-age” disease with an “age-old” solution. Cancer Lett.

[30] Dhule SS, Penfornis P, Frazier T, Walker R, Feldman J, Tan G, He J, Alb A, John V, Pochampally R (2012). Curcumin-loaded gamma-cyclodextrin liposomal nanoparticles as delivery vehicles for osteosarcoma. Nanomed: Nanotechnol, Biol Med.

[31] Dhule SS, Penfornis P, He J, Harris MR, Terry T, John V, Pochampally R (2014). The combined effect of encapsulating curcumin and C6 ceramide in liposomal nanoparticles against osteosarcoma. Mol Pharmacol.

[32] Saengkrit N, Saesoo S, Srinuanchai W, Phunpee S, Ruktanonchai UR (2014). Influence of curcumin-loaded cationic liposome on anticancer activity for cervical cancer therapy. Colloids Surf B: Biointerfaces.

[33] Huang Q, Zhang L, Sun X, Zeng K, Li J, Liu Y-N (2014). Coating of carboxymethyl dextran on liposomal curcumin to improve the anticancer activity. RSC Adv.

[34] Li L, Ahmed B, Mehta K, Kurzrock R (2007). Liposomal curcumin with and without oxaliplatin: effects on cell growth, apoptosis, and angiogenesis in colorectal cancer. Mol Cancer Ther.

[35] Pradhan P, Giri J, Rieken F, Koch C, Mykhaylyk O, Doblinger M, Banerjee R, Bahadur D, Plank C (2010). Targeted temperature sensitive magnetic liposomes for thermo-chemotherapy. J Control Release.

[36] Kuo C-Y, Liu T-Y, Hardiansyah A, Chiu W-Y: Magnetically polymeric nanocarriers for targeting delivery of curcumin and hyperthermia treatments toward cancer cells. J. Polym. Sci A Polym Chem. 2016:n/a-n/a.

[37] Hasan M, Belhaj N, Benachour H, Barberi-Heyob M, Kahn CJ, Jabbari E, Linder M, Arab-Tehrany E (2014). Liposome encapsulation of curcumin: physico-chemical characterizations and effects on MCF7 cancer cell proliferation. Int J Pharm.

[38] Rejinold NS, Muthunarayanan M, Divyarani VV, Sreerekha PR, Chennazhi KP, Nair SV, Tamura H, Jayakumar R (2011). Curcumin-loaded biocompatible thermoresponsive polymeric nanoparticles for cancer drug delivery. J Colloid Interface Sci.

[39] Chen Y, Wu Q, Zhang Z, Yuan L, Liu X, Zhou L (2012). Preparation of curcumin-loaded liposomes and evaluation of their skin permeation and pharmacodynamics. Molecules.

[40] Yallapu MM, Othman SF, Curtis ET, Bauer NA, Chauhan N, Kumar D, Jaggi M, Chauhan SC (2012). Curcumin-loaded magnetic nanoparticles for breast cancer therapeutics and imaging applications. Int J Nanomedicine.

[41] Bloemen M, Brullot W, Luong TT, Geukens N, Gils A, Verbiest T (2012). Improved functionalization of oleic acid-coated iron oxide nanoparticles for biomedical applications. J Nanopart Res.

[42] Ding X, Cai K, Luo Z, Li J, Hu Y, Shen X (2012). Biocompatible magnetic liposomes for temperature triggered drug delivery. Nanoscale.

[43] Lu Z, Prouty MD, Guo Z, Golub VO, Kumar CSSR, Lvov YM (2005). Magnetic switch of permeability for polyelectrolyte microcapsules embedded with Co@Au nanoparticles. Langmuir.

[44] Pradhan P, Giri J, Banerjee R, Bellare J, Bahadur D (2007). Preparation and characterization of manganese ferrite-based magnetic liposomes for hyperthermia treatment of cancer. J Magn Magn Mater.

[45] Béalle G, Di Corato R, Kolosnjaj-Tabi J, Dupuis V, Clément O, Gazeau F, Wilhelm C, Ménager C (2012). Ultra magnetic liposomes for MR imaging, targeting, and hyperthermia. Langmuir.

[46] Syng-Ai C, Kumari AL, Khar A (2004). Effect of curcumin on normal and tumor cells: role of glutathione and bcl-2. Mol Cancer Ther.

